# Significance of Mitochondrial Dynamics in Reproductive Physiology: Current and Emerging Horizons in Mitochondrial Therapy for Assisted Reproductive Technologies

**DOI:** 10.1002/rmb2.12672

**Published:** 2025-08-17

**Authors:** Sanath Udayanga Kankanam Gamage, Yoshiharu Morimoto

**Affiliations:** ^1^ HORAC Grand Front Osaka Clinic Osaka Japan

**Keywords:** ascent, infertility, mitochondria, mitochondrial dysfunction, mitochondrial therapies for infertility, mitochondrial transplantation, reproductive aging

## Abstract

**Background:**

Mitochondria play a critical role in cellular bioenergetics and signaling, with particular importance in the context of reproductive biology. This review summarizes their role in reproduction and explores current and emerging mitochondrial therapies for fertility treatment.

**Methods:**

A comprehensive literature search using terms like mitochondria, infertility, reproduction, gametes, mitochondrial replacement, and mitochondrial transplantation identified relevant studies on mitochondria's role in gametogenesis, fertilization, and early embryonic development in relevant databases. Selected publications were reviewed and summarized to present current and future mitochondrial therapies for fertility.

**Main Findings:**

Mitochondrial dynamics and functions are critical for meeting the energy requirements of essential reproductive processes, including gametogenesis, fertilization, and early embryonic development. Dysregulation of mitochondrial function has been associated with a range of reproductive disorders, such as infertility, recurrent pregnancy loss, and maternally inherited mitochondrial diseases. Emerging therapeutic strategies, such as mitochondrial replacement therapy, antioxidant supplementation, and mitochondrial transplantation, offer promising avenues for overcoming these challenges and improving reproductive outcomes.

**Conclusions:**

Utilizing mitochondrial‐based therapies represents a promising and innovative approach in the advancement of fertility treatments. Ongoing research and clinical development in this area hold significant potential to enhance reproductive outcomes and improve the quality of life for individuals and couples facing fertility challenges.

## Graphical abstract

Mitochondria are crucial for reproductive processes, and their dysfunction is implicated in infertility and related disorders. This review examines the role of mitochondria in reproduction and explores emerging therapies. Promising therapies including antioxidants and mitochondria transplantation may offer potential advancements in fertility treatment.

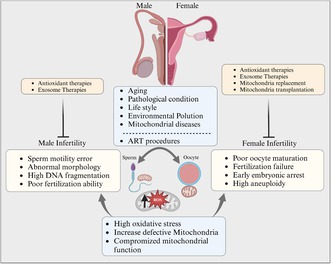

## 1 Introduction

Mitochondria are remarkable subcellular organelles that play a crucial role in the bioenergetic processes within eukaryotic cells through oxidative phosphorylation [1, 2, 3, 4]. The mitochondria derived from an alpha‐proteobacterial ancestor and have evolved to become one of the two endomembrane systems in non‐photosynthetic eukaryotes, along with the endoplasmic reticulum [5, 6]. Beyond their energetic role, mitochondria are crucial for various cellular processes, including calcium homeostasis, redox signaling, and apoptosis [7, 8, 9, 10]. The mitochondria exhibit remarkable adaptability, constantly adjusting their morphology, number, size, movement, and location within cells to seamlessly integrate their function with the cell's physiological shifts [11]. This mitochondrial dynamism underscores their functional versatility, extending beyond static energy production to encompass metabolic regulation through continuous fission and fusion cycles in response to cellular demands [12]. These processes are essential for fulfilling various physiological functions and are indispensable for maintaining a healthy mitochondrial network and cellular homeostasis [13, 14]. In general, the dynamic nature of mitochondria allows them to adapt to changing cellular conditions and respond to stress conditions. Thus, mitochondrial dynamics are vital for maintaining the health, function, and distribution of mitochondria in reproductive cells.

The tricarboxylic acid (TCA) cycle, a crucial metabolic pathway within the mitochondrial matrix, oxidizes carbohydrates, fats, and amino acids to generate reducing equivalents (Nicotinamide adenine dinucleotide (NADH) and Flavin adenine dinucleotide (FADH2)), which fuel the electron transport chain (ETC) for ATP production [15]. Pyruvate from glycolysis is converted into acetyl‐CoA, which enters the TCA cycle, producing key intermediates like α‐ketoglutarate and succinate that contribute to biosynthesis and cellular signaling [16]. Beyond energy production, TCA cycle metabolites regulate chromatin modifications, DNA methylation, and protein modifications, influencing cell differentiation, immune function, and stress responses. Dysregulation of this cycle is linked to infertility, cancer, and metabolic disorders, making it a potential therapeutic target [15]. The ETC, embedded in the inner mitochondrial membrane, utilizes electrons from NADH and FADH2 to establish a proton gradient, driving ATP synthase to catalyze ATP formation through oxidative phosphorylation [17]. This efficient process couples electron transfer with ATP generation, sustaining essential cellular activities.

Moreover, mitochondria possess their own distinct DNA separate from the nuclear genome [18, 19]. The mitochondrial DNA (mtDNA) is a circular molecule encoding 37 genes crucial for mitochondrial function, including energy production through oxidative phosphorylation [20]. Unlike nuclear DNA, which is inherited from both parents, mtDNA is primarily inherited maternally [21, 22]. The small size and high copy number of mtDNA, along with its exposure to reactive oxygen species generated during oxidative phosphorylation, make it more susceptible to mutations compared to nuclear DNA [23]. While most mtDNA mutations are benign, some can disrupt mitochondrial function, leading to a range of diseases affecting energy‐demanding organs like the brain, heart, muscles, and reproductive competence and outcomes [2, 24].

In the context of reproductive biology, the complex relationship between mitochondrial function and cellular physiology is particularly relevant in the framework of reproductive biology and fertility [2, 25]. The importance of mitochondria is further amplified due to the high energy demands for gametogenesis, fertilization, and early embryonic development [1, 26, 27, 28, 29, 30, 31]. These energy‐intensive processes underscore the critical importance of a robust and adaptable mitochondrial network. The capacity for rapid mitochondrial adaptation to dynamic cellular conditions is not only fundamental for normal cellular function but also plays a pivotal role in successful embryo development [30]. Mitochondrial dysfunction has been implicated in various reproductive disorders, including reducing the quality of gamete cells, recurrent miscarriage, and inherited mitochondrial diseases [28, 29, 32].

This review investigates the complex interplay between mitochondrial dynamics and function in the context of reproductive health, encompassing both male and female perspectives. We begin with the dynamics of mitochondria within sperm cells, oocytes, cumulus cells, and early embryos, providing a comprehensive understanding of their function at each stage. Recognizing that mitochondrial dysfunction can have detrimental effects on reproductive outcomes, we then explore the factors that can compromise mitochondrial function and dynamics in the reproductive cells. We explore the available therapeutic approaches to improve the mitochondria and their benefits and disadvantages. Looking towards the future, we discuss the emerging field of mitochondrial transplantation therapies, focusing on their existing and potential applications in reproductive medicine. Specifically, we highlight the potential of stem cell mitochondria as a promising new avenue for reproductive therapies.

## 2 Mitochondria Biogenesis and Dynamics in Relation With Reproductive Physiology

**FIGURE 1:**
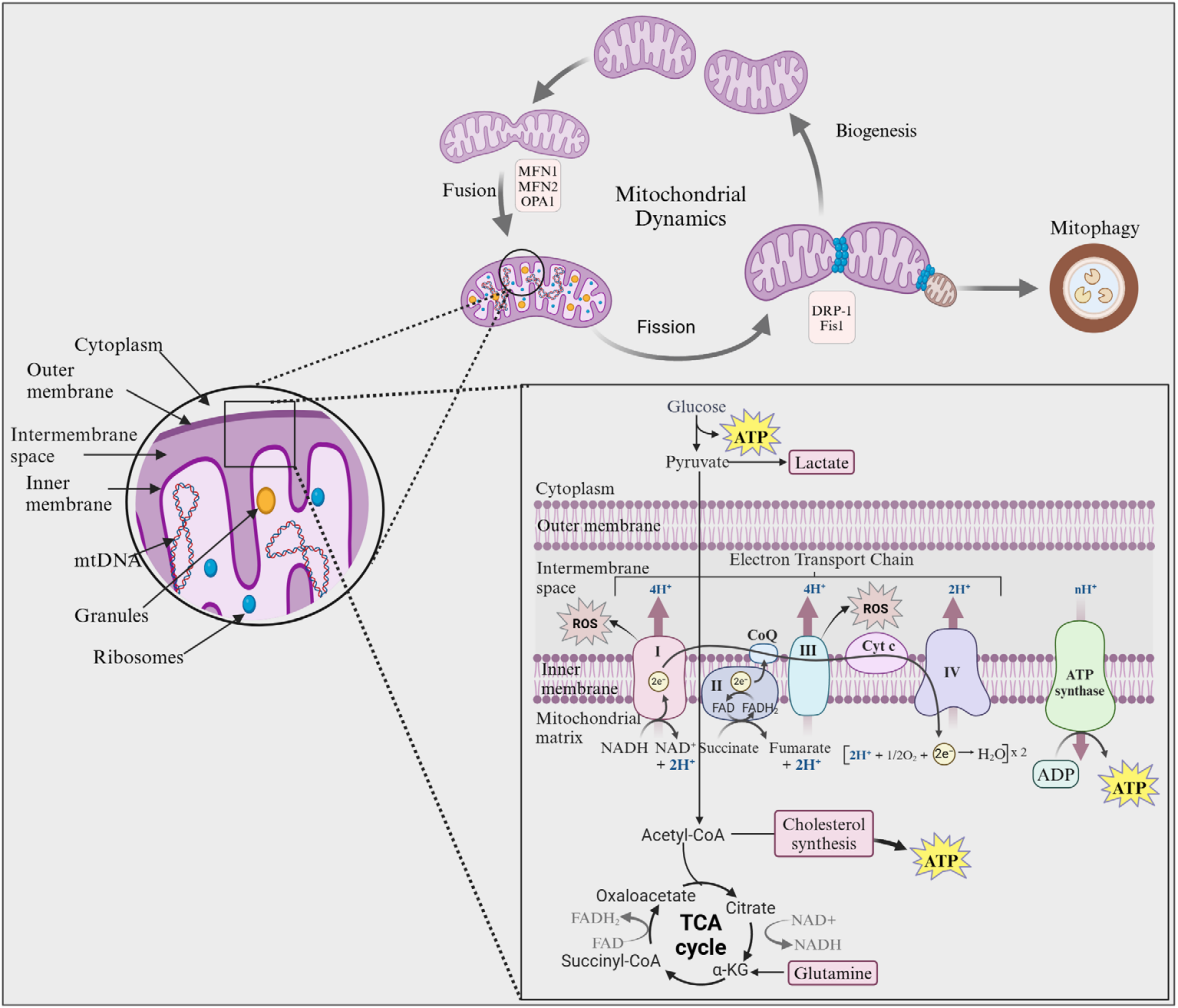
Mitochondria dynamics, biogenesis, and metabolism. Interplay of mitochondrial dynamics, biogenesis, and metabolism. Mitochondrial morphology is dynamically regulated by fusion and fission, biogenesis replenishes the mitochondrial pool. These processes are integrated with mitochondrial metabolism, where the TCA cycle and electron transport chain generate signaling molecules that influence cellular function.

Mitochondrial biogenesis is a complex process by which cells increase their mitochondrial mass, involving the coordinated expression of nuclear and mitochondrial genes encoding mitochondrial proteins and other molecules. This tightly regulated process is essential for meeting cellular energy demands, maintaining metabolic homeostasis, and adapting to various physiological stimuli and stresses, particularly in energy‐intensive processes like reproduction. The biogenesis involves a tightly coordinated interplay between nuclear and mitochondrial genomes, orchestrating the synthesis, import, and assembly of over 1000 proteins that constitute a functional mitochondrion [33]. Increased energy demand, ATP, and cellular redox state trigger signaling pathways that activate transcription factors, such as peroxisome proliferator‐activated receptor gamma coactivator 1‐alpha (PGC‐1α) [15]. Further, mitochondrial biogenesis not only increases the number of mitochondria but also enhances the expression and activity of enzymes involved in the TCA cycle and oxidative phosphorylation, which represent a tightly interwoven symphony of cellular energetics (Figure 1) [34]. And the therapeutic strategies aimed at enhancing mitochondrial biogenesis may offer promising avenues for improving fertility outcomes in individuals experiencing infertility related to mitochondrial dysfunction.

In accordance, the mitochondria are constantly undergoing transformation through processes of fusion and fission, which are reflected in the morphological changes of mitochondrial cristae and the extension or fragmentation of the tubular network. Mitochondrial fusion proteins mitofusin 1 (Mfn1) & mitofusin 2 (Mfn2) facilitate the merging of the inner and outer membranes, creating a unified mitochondrial network that ensures mitochondrial quality. The GTPase Optic Atrophy 1 supports the fusion of the inner membrane and maintains the integrity of the cristae structure. Dynamin‐related protein 1 (DRP1), a key player in mitochondrial division, enables rapid shifts in division activity based on the cell's requirements. The oocyte‐specific deletion of Drp1 results in the accumulation of abnormally fused mitochondria, disrupted calcium oscillations, impaired secretory function, and defective meiosis [35]. Also, inhibition of DRP1 using Mdivi‐1 has been shown to impair blastocyst formation in pigs, characterized by reduced mitochondrial membrane potential and elevated ROS levels [36]. Research indicates that the absence of Mfn1 and Mfn2 in mouse oocytes leads to infertility and loss of the follicular reserve, while DRP1 expression decreases in aging oocytes [37]. Similarly, oocytes with a knockout of Mfn1 exhibit severe mitochondrial structural abnormalities, including disrupted cristae and reduced matrix density, leading to developmental arrest and impaired oocyte–granulosa cell communication [38]. Knockout of Mfn2 alone in mouse oocytes results in reduced maturation and fertilization rates, altered mitochondrial distribution, and abnormal spindle morphology, highlighting the importance of mitochondrial dynamics in chromosome segregation during meiosis [39]. Further, siRNA‐mediated knockdown of Mfn2 significantly decreases blastocyst formation and the progression of embryos beyond the third cell division, suggesting a critical role for mitochondrial fusion during early embryonic genome activation [40, 41].

Conversely, overexpression of Mfn1 or Mfn2 results in irregular mitochondrial distribution, mitochondrial aggregation, and excessive contact between mitochondria and the endoplasmic reticulum (ER). These abnormalities lead to disrupted calcium homeostasis and erroneous chromosomal segregation during meiosis [42]. Therefore, both deficiency and overexpression of mitochondrial dynamics‐related proteins can result in morphological mitochondrial defects, dysregulated activity, increased ROS production, and disturbed intracellular signaling pathways [43].

### 2.1 Gametogenesis

Gametogenesis, the intricate process of generating specialized haploid cells from primordial germ cells known as gametes, sperm and oocytes, is fundamental for sexual reproduction. This process involves a tightly orchestrated series of cellular events, including mitotic proliferation, meiotic division, and extensive cellular differentiation. Throughout these transformative stages, mitochondrial metabolism plays a pivotal role in providing the energy and biosynthetic precursors essential for successful gamete formation.

During gametogenesis, the energy demands placed upon germ cells are particularly high, as they undergo rapid proliferation, intricate chromosomal rearrangements, and extensive cytoplasmic remodeling [44]. Mitochondria in primordial germ cells exhibit a rounded morphology (> 0.6 μm in diameter) with tubulo‐vesicular cristae [45]. Dysfunctional mitochondria or impaired mitochondrial metabolism can severely compromise gametogenesis, leading to infertility, developmental defects in offspring, and even embryonic lethality [46].

#### 2.1.1 Spermatogenesis

Spermatogenesis, the complex and tightly regulated process of sperm cell development, requires a significant energy supply to produce mature, functional spermatozoa [47]. Mitochondrial biogenesis, encompassing the coordinated synthesis of mitochondrial proteins and lipids for organelle assembly, and the resultant ATP production are essential for supporting energy‐intensive cellular processes such as DNA replication, transcription, translation, and cellular differentiation [47]. As spermatogonial stem cells differentiate into spermatocytes and then spermatids, the demands for ATP production increase dramatically; to meet these high energy requirements, the number of mitochondria in developing sperm cells must expand through mitochondrial biogenesis [47, 48]. Further, increased expression of MFN1 and DRP1 in differentiating spermatogonia [49] precisely coordinates the mitochondrial fusion and fission to support spermatogonial differentiation. Therefore, the mitochondrial network within sperm cells undergoes dynamic changes during spermatogenesis and epididymal maturation [25, 48] which is significant for the quality of the sperm.

Disruptions in mitochondrial biogenesis and dynamism during spermatogenesis can have detrimental impacts on sperm quality and quantity, contributing to male infertility. Impaired mitochondrial biogenesis during the spermatogenesis process may lead to an inadequate number of functional mitochondria in mature sperm, compromising their ability to generate sufficient ATP for motility, capacitation, and the acrosome reaction all—crucial steps for successful fertilization [50, 51]. Additionally, normal mitochondrial respiration is required for mammalian spermatogenesis, and mitochondrial dysfunction arising from disrupted biogenesis can increase oxidative stress (OS) and damage to sperm mtDNA, further impairing sperm function and fertility [52]. Understanding the mechanisms regulating mitochondrial biogenesis and dynamism is, therefore, an important area of study for addressing mitochondrial‐related causes of male infertility.

#### 2.1.2 Oogenesis

Upon differentiation into oogonia, mitochondria get slightly enlarge (0.8–1.0 μm in diameter) and develop sparse, lamellar cristae [45]. Subsequent oocyte differentiation leads to a conventional mitochondrial configuration, characterized by an elongated shape (1.2–1.9 μm in length), numerous transversely oriented cristae, and a single large vacuole [53]. These morphological changes suggest an increased capacity for oxidative phosphorylation. However, this configuration is temporary, and dumbbell‐shaped mitochondria become prevalent in early developing oocytes, eventually transforming into round/oval vacuolated organelles with columnar‐shaped, arched cristae [53]. The increasing interplay observed between mitochondria, smooth endoplasmic reticulum, and lipid droplets during oogenesis suggests a crucial functional role for the mitochondria network in the later stages of oocyte development [54, 55, 56].

During the oogenesis, immature oocytes avoid the damaging effects of reactive oxygen species (ROS) by suppressing complex I, a key generator of ROS in the mitochondrial electron transport chain. By eliminating complex I, oocytes maintain a low‐ROS environment, ensuring cellular fitness and longevity, which is essential for their long dormancy while developing an oocyte [57]. Interestingly, despite the absence of complex I, mitochondria in early oocytes remain functionally active in terms of ATP synthesis, although with lower activity. In addition, antioxidant defense mechanisms based on superoxide dismutase 1 (SOD1) are prominent in dormant oocytes in ovaries to protect the immature oocytes and to maintain low ROS levels [58]. Mitochondrial dynamics during spindle formation and meiosis I ensure optimal mitochondrial positioning for spindle function while preserving oocyte mitochondrial reserves at minimal activity levels [59]. This unique mitochondrial adaptation and precautionary strategies allow oocytes to balance essential metabolic processes while preventing ROS accumulation.

Moreover, some subcellular protein, mitochondrial ubiquitin ligase (MARCH5) that co‐localizes with the mitochondria during mouse oocyte meiotic progression, regulates mitochondrial function and/or ubiquitination of microtubule dynamics or cell cycle‐regulating proteins [60]. Some research demonstrated that mitochondrial dysfunction can disrupt the pairing of homologous chromosomes during meiosis [61]. This abnormality is potentially linked to the inhibition of mitochondrial function caused by the deletion of spindle defective protein 3 on the outer mitochondrial membrane [61]. Thus, mitochondrial dynamics and function have significant implications during oogenesis and meiotic maturation.

#### 2.1.3 Mature Spermatozoa and Mitochondria

Mature spermatozoa rely heavily on mitochondria to fuel their motility. The midpiece of mature spermatozoa is densely packed with mitochondria, which generate ATP through oxidative phosphorylation to fuel the flagellar movement required for sperm to traverse the female reproductive tract and reach the egg [62, 63]. Further, mitochondria play a crucial role in regulating apoptosis, a critical process for eliminating defective sperm cells during spermatogenesis to ensure the production of high‐quality gametes capable of successful fertilization [47].

**FIGURE 2:**
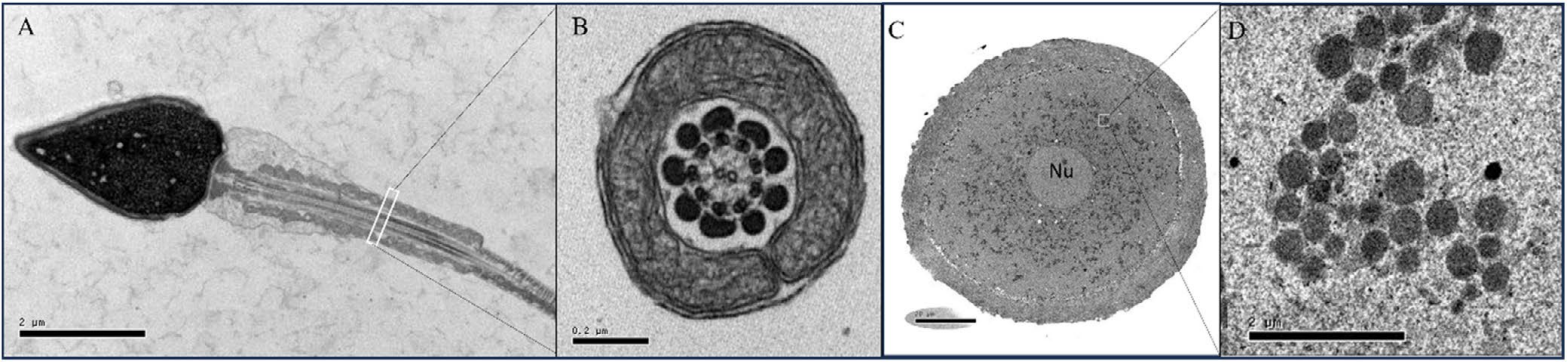
Transmission electron microscope figure of human sperm and oocyte. (A) Human sperm cell with packed mitochondria sheath in the mid‐piece, scale bar‐2 μm. (B) Enlarged cross section of the midpiece of the human sperm cell, elongated mitochondria tightly packed around the axoneme, scale bar‐0.2 μm. (C) Metaphase I oocyte showing homogeneous mitochondrial distribution throughout the cytoplasm. N‐Nucleus. Scale bar‐20 μm. (D) Enlarged piece of cytoplasm, showing plenty of spherical‐shaped mitochondria (dark black), scale bar‐2 μm.

Imbalances in mitochondrial function, such as altered redox status and calcium homeostasis, can trigger the intrinsic apoptotic pathway, leading to sperm death and impaired fertility [26, 63]. Sperm mtDNA is particularly susceptible to damage from oxidative stress due to its close proximity to the sites of reactive oxygen species production within the mitochondria (Figure 2A,B), as well as the limited DNA repair mechanisms present in the mitochondria compared to the nuclear genome [63]. Studies have shown that increased levels of mtDNA damage and mutations in sperm are correlated with poor semen quality, including decreased sperm motility, abnormal morphology, and higher rates of DNA fragmentation [62, 64]. The integrity of the sperm mtDNA is critical for maintaining proper mitochondrial function [52], which is essential for powering sperm motility, capacitation, and the acrosome reaction, all critical steps for successful fertilization and the establishment of successful embryo development.

#### 2.1.4 Mature Oocyte and Mitochondria

Oocytes as the largest cells in the human body, rely heavily on mitochondria for energy storage, cytoplasmic maturation, and calcium homeostasis. In fully developed oocytes, mitochondria maintain a round/oval shape (0.4–0.6 μm) with a large vacuole and arched/concentrically arranged cristae (Figure 2C,D) [53, 56]. In the GV stage, mitochondria in oocytes were evenly distributed throughout the ooplasm [65]. Interestingly, in ruminant oocytes, mitochondria adopt a unique hooded morphology, the functional implications of which remain unclear [66]. As oocytes progressed to the MI and MII stages, mitochondria aggregated and formed clusters; the mean size of mitochondrial clusters and the proportions of clustered mitochondria increased along with the maturation of oocytes [65]. Mitochondrial biogenesis and distribution within the oocyte are tightly regulated to ensure the proper segregation of these organelles into the mature oocyte, which will subsequently fuel early embryonic development [1, 2, 67]. Studies have shown that the quantity and distribution of mitochondria within the oocyte are crucial determinants of the cell's developmental competence, ensuring the oocyte is equipped with the sufficient energy reserves, necessary proteins, mRNA transcripts, and nutrients to support early embryonic development [68, 69].

They also play a vital role in regulating Ca^2+^ levels within the oocyte, which is essential for key events in oocyte maturation, such as meiotic spindle formation, chromosome segregation, and polar body extrusion, as well as for the initiation of embryonic development following fertilization [70, 71]. Disruptions in mitochondrial calcium handling can have detrimental effects on these critical processes, leading to impaired oocyte quality and developmental competence [27]. The mitochondrial calcium uniporter (MCU) protein facilitates the rapid influx of Ca^2+^ into mitochondria, ensuring sufficient energy supply during meiosis. Studies have revealed that specific deletion of MCU results in low mitochondrial Ca^2+^ concentrations, leading to low ATP levels, abnormal spindle assembly, and disrupted meiosis progression [72]. Conversely, overactivation of AMP‐activated protein kinase (AMPK) and Ca^2+^ overload can trigger oxidative stress, apoptosis, and meiotic cell cycle arrest [73, 74].

The dynamic mitochondrial changes during oocyte maturation are essential for the development of a high‐quality, fertilization‐competent egg. Throughout this process, mitochondria undergo dynamic active redistribution, moving towards regions of high energy demand within the oocyte, including the developing meiotic spindle and the cortical region [11, 27]. Mitochondria are strategically positioned around the meiotic spindle, providing ATP for microtubule dynamics and chromosome movement during meiosis [24]. The proper distribution and function of mitochondria are essential for the formation of a well‐organized meiotic spindle, ensuring accurate chromosome segregation and the successful completion of meiosis, which is critical for the production of a developmentally competent oocyte [11, 32]. This strategic positioning of mitochondria ensures a localized supply of ATP to support critical processes like spindle formation and chromosome segregation [30, 67].

Moreover, mtDNA plays a crucial role in oocyte quality and embryonic development due to its unique inheritance pattern and essential function in cellular energy production. A sufficient number of healthy mitochondria, each containing multiple copies of mtDNA, are crucial for providing the energy required for oocyte maturation, fertilization, and early embryonic development [2]. Unlike nuclear DNA, which is inherited from both parents, mtDNA is exclusively maternally inherited, where it is passed down from the mother to her offspring through the oocyte. This maternal inheritance pattern makes mtDNA particularly vulnerable to the accumulation of mutations over generations, potentially impacting oocyte quality and offspring health [75, 76, 77]. Within the oocyte, mtDNA encodes essential components of the electron transport chain, responsible for generating ATP through oxidative phosphorylation [29]. mtDNA mutation and depletion have been associated with poor oocyte quality, impaired fertilization, and compromised embryo development, highlighting the critical [2, 24] role of the mitochondria in reproductive competence [25]. Disruptions in any of these mitochondrial dynamics during oocyte maturation can have significant consequences for oocyte quality and developmental competence, leading to increased chromosomal abnormalities, fertilization failure, and early embryonic arrest [11, 27, 68].

### 2.2 Cumulus Cell and Mitochondria

Cumulus cells count on mitochondrial function to support the energy‐intensive processes of oocyte maturation and ovulation. Mitochondrial dysfunction in cumulus cells has been associated with poor oocyte quality and reduced fertilization rates [78]. The mitochondrial activity and metabolic function of cumulus cells have been directly linked to oocyte quality and developmental competence. Studies have shown that cumulus cell mitochondrial function, as measured by ATP production and oxygen consumption, is a strong predictor of oocyte quality and embryo developmental potential [78, 79, 80]. The gamete is maintained in a relatively quiescent metabolic state, characterized by reduced catabolic activity, due to the provision of energetic substrates by cumulus cells and granulosa cells, including ATP, pyruvate, NADPH, cholesterol, and specific amino acids [81, 82, 83, 84].

A recent study revealed the link between granulosa cell metabolism and oocyte developmental competence, particularly in the context of obesity and aging using a mouse model and a human cohort undergoing in vitro fertilization or intracytoplasmic sperm injection (IVF/ICSI) [85]. The cumulus metabolism undergoes dynamic changes in response to the LH surge and high cumulus cell respiration correlated with failed fertilization in a subset of women, while glycolytic reserve and mitochondrial ATP production were positively associated with successful blastocyst development. Thus, the cumulus cell metabolism plays a crucial role in oocyte quality and is significantly impacted by age and obesity, potentially contributing to female infertility [85].

Understanding the dynamics of mitochondrial function in these processes, particularly the role of cumulus cell mitochondria, may be necessary for addressing infertility and improving reproductive outcomes. Given the close relationship between cumulus cell mitochondrial function and oocyte quality, cumulus cell mitochondria are being explored as a potential non‐invasive biomarker for assessing oocyte quality and predicting embryo viability [86].

## 3 Mitochondria and Embryo Development

Mitochondrial function is intimately involved in the complex process of sperm‐egg membrane fusion during fertilization. The release of reactive oxygen species from sperm mitochondria is thought to play a crucial role in triggering the acrosome reaction, which exposes the sperm's hydrolytic enzymes required for penetrating the egg's zona pellucida [87, 88]. Additionally, mitochondrial‐derived ATP powers the structural rearrangements and signaling cascades that enable the fusion of the sperm and egg membranes [88].

Upon fertilization, the sperm's mitochondria are selectively degraded [89, 90, 91], leaving the oocyte's mitochondria as the sole source of mtDNA for the developing embryo [30]. The selective elimination of paternal mitochondria ensures that the embryo inherits a homogeneous population of mitochondria from the mother, which is critical for maintaining mitochondrial function and preventing mitochondrial DNA diseases [92, 93]. This maternal inheritance pattern highlights the critical importance of oocyte mitochondria for successful fertilization and early embryonic development. Disruptions in mitochondrial function or mtDNA integrity can have devastating consequences, leading to impaired embryo development, chromosome segregation errors, and an increased risk of developmental abnormalities [94, 95].

Oocyte mitochondria provide the necessary energy and signaling functions to support the rapid cell divisions and cellular differentiation that occur during the initial stages of embryogenesis [28, 68]. Further, mitochondria fission protein, DRP1, plays a crucial role in the cell signaling pathways that regulate gene expression and determine cell fate during the early stages of embryonic development [96].

Interestingly, new mitochondrial biogenesis is suppressed during the initial cleavage divisions of the early embryo. Instead, the existing maternal mitochondrial pool inherited from the oocyte is evenly distributed among the daughter cells, ensuring each blastomere receives an adequate number of mitochondria to support their energy needs [97]. Between the zygote and blastocyst stages, mtDNA replication is halted, leading to a reduction in mtDNA copy number during early embryogenesis due to dilution from rapid cell divisions. This temporary mitochondrial replication pause allows the embryo to focus its resources on rapid cell division rather than mitochondrial expansion [98]. Later in embryonic development, once the embryonic genome is activated, mitochondrial replication resumes to meet the increasing energy demands of cellular differentiation and growth [99, 100, 101]. Experimental findings in rhesus monkeys and mice suggest the presence of an additional genetic bottleneck during this early embryonic window, occurring prior to the reinitiation of mtDNA replication [102, 103]. This reactivation of mitochondrial biogenesis ensures the embryo has a sufficient mitochondrial population to support the diverse cellular functions required during organogenesis and fetal development [31, 104].

During the initial stages of human preimplantation embryo development, a dynamic shift in mitochondrial function and DNA replication can be found, and there is a strong interaction between mitochondrial quantity in the timeline of development, particularly the timing of expanded blastocyst formation [101, 105, 106]. The pattern of mitochondrial membrane potential progressively changes throughout preimplantation development, and there is a correlation between the increasing complexity of the developing embryo and the escalating respiratory function of its mitochondria, evidenced by heightened oxygen consumption rates and cytochrome C oxidase activity [101, 107]. However, contrary to the observed increase in mitochondrial function, the study revealed a transient decrease in mitochondrial DNA copy number before blastulation, the stage at which the embryo forms a hollow sphere of cells. Moreover, the existing mitochondria might be undergoing functional changes and achieving higher energy output even with a lower copy number of their DNA [101]. Furthermore, the mitochondrial dynamics regulate embryonic development through a mechanism that maintains Ca^2+^/calmodulin‐dependent protein kinase II homeostasis and stabilizes β‐Catenin protein [108]. These evidences confirm that mitochondrial dynamic behavior has valuable insights into the intricate processes and the governance of early embryo development.

## 4 Mitochondrial Dysfunction and Challenges Reproductive Health

Impaired mitochondrial function in gametes or embryos can have significant consequences for reproductive health, leading to infertility, miscarriage, and birth defects. Factors such as aging, obesity, diabetes, environmental pollution, and genetic mutations can contribute to mitochondrial dysfunction [29]. Additionally, mitochondrial dysfunction can also lead to chromosomal abnormalities, impairing meiotic division and increasing the risk of aneuploidy in the resulting embryo [29, 109]. Furthermore, the mtDNA mutations and defects have been associated with poor oocyte quality, impaired fertilization, and compromised embryo development [29, 109].

**FIGURE 3:**
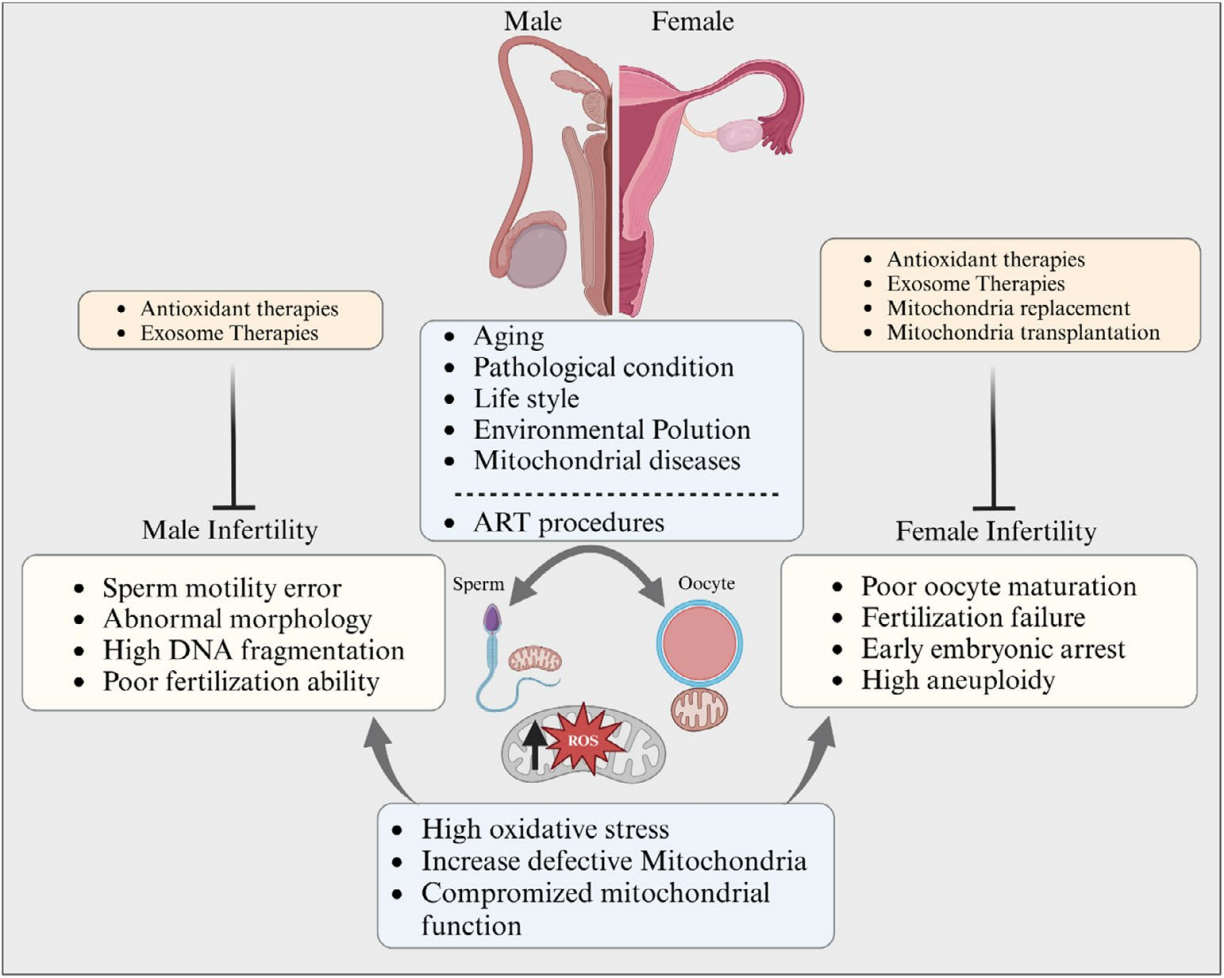
Schematic representation of factors compromising gamete mitochondrial function and resultant consequences, along with current therapeutic interventions for infertility related to these conditions.

Understanding the intricate mechanisms by which mitochondria contribute to these processes is essential for developing strategies to address infertility, prevent the transmission of mitochondrial disorder related defects, and enhance reproductive health. To address these challenges, researchers have explored various mitochondrial therapies, including the use of mitochondrial chemical supplements, mitochondrial replacement, mitochondrial transfer, and gene therapy (Figure 3). These emerging technologies aim to enhance mitochondrial function and improve reproductive outcomes for individuals struggling with infertility.

### 4.1 Oxidative Stress and Mitochondrial Functions

Oxidative stress (OS) is an imbalance between reactive oxygen species (ROS) and antioxidants; it disrupts mitochondrial dynamics, leading to mitochondrial fragmentation, impaired fusion, defective mitophagy, and altered inter‐organelle communication, which collectively contribute to cellular dysfunction and disease progression [110]. OS is shown to adversely impact gametes and embryos, compromising outcomes such as fertilization, embryo development, and pregnancy success [111]. The key ROS sources include both internal cellular processes within sperm, oocytes, and embryos due to the physiological conditions of patients, as well as external factors like ART setting and environmental conditions. This oxidative damage can impair mitochondrial DNA integrity, disrupt electron transport chain function, and trigger mitochondrial‐mediated apoptosis by letting the release of cytochrome C, ultimately compromising reproductive cell functions.

Excessive ROS has been shown to induce lipid peroxidation in sperm membranes, which can impair mitochondrial dynamics, sperm motility, and reduce the sperm's ability to undergo the acrosome reaction, a crucial step in fertilization [63]. Additionally, ROS‐induced DNA damage in sperm cells has been linked to increased rates of miscarriage and congenital abnormalities in the resulting offspring [62, 112]. Thus, high OS can lead to reduced motility, DNA fragmentation, and impaired fertilization capacity.

In oocytes, OS can disrupt meiotic spindle formation, impair chromosome segregation, and compromise fertilization and embryo development [113, 114]. Further, during meiotic prophase, OS can lead to chromosome segregation errors and may play a role in the loss of cohesion in aging human oocytes [115]. Oocytes, particularly during aging, experience increased ROS generation due to mitochondrial dysfunction and loss of AMP‐activated protein kinase activity [116, 117]. mtDNA damage further impairs mitochondrial function, creating a vicious cycle of increased ROS and declining oocyte quality [118].

Moreover, even though assisted reproductive technologies (ART), such as IVF and ICSI, have revolutionized infertility treatment, certain ART procedures, including laboratory oxygen levels, CO_2_ incubators, temperature, humidity, and cryopreservation, can induce cellular stress and potentially affect mitochondrial function in gametes and early embryos [2, 24]. Recent studies have shown that the cryopreservation and thawing process during IVF can lead to increased OS and impaired mitochondrial function in human oocytes, potentially reducing their developmental competence [109, 119, 120]. Prolonged in‐vitro maturation (IVM) reduces the developmental rate of bovine oocytes to blastocysts, altering mitochondrial dynamics [121]. Mitochondrial copy number, membrane potential, and ATP content are decreased in murine oocytes following controlled ovarian hyperstimulation (COH) [122] and COH causes mitochondrial abnormalities in granulosa cells of Rhesus monkeys [123]. And, factors such as gamete manipulation, culture media composition, and the in vitro environment can contribute to mitochondrial dysfunction in reproductive cells [24, 63, 111]. Additionally, the culture conditions of embryos in vitro during IVF may expose them to suboptimal oxygen levels and nutrient availability, leading to OS and mitochondrial dysfunction [124]. These stressors can trigger lipid peroxidation, DNA fragmentation, and apoptotic pathways, especially within ART settings where ROS levels often exceed physiological norms.

In total, OS resulting from technical manipulations during ART procedures may exert a significant impact on mitochondrial integrity in gametes and early embryos, potentially leading to defects in offspring. Therefore, understanding the impact of ART techniques on mitochondrial function and dynamics in gametes and early embryos is a key determinant of developing strategies to mitigate these challenges and improve reproductive outcomes. Optimizing culture conditions, refining sperm selection methods, and potentially supplementing with mitochondrial‐targeted therapies may help address the mitochondrial dysfunction associated with ART procedures.

### 4.2 Pathological and Physiological Conditions and Mitochondrial Functions

#### 4.2.1 Diabetes Mellitus

Oxidative stress and chronic inflammation are critical factors in the pathogenesis and progression of diabetes and its associated complications, and both can impair mitochondrial function in sperm and oocytes [125, 126, 127]. Diabetes disrupts this delicate balance of fission and fusion, mitochondrial dynamics tilting the scales towards excessive mitochondrial fission, leading to fragmented mitochondria with impaired energy production and increased susceptibility to apoptosis [26, 128]. Hyperglycemia and insulin resistance in diabetes can lead to excessive reactive oxygen species production, which can damage mtDNA and disrupt mitochondrial bioenergetics in reproductive cells in both genders [126].

In males, the oxidative damages mitochondrial membranes, disrupt electron transport chain activity, and impair ATP production, compromising sperm motility, morphology, and viability [126, 129]. Both type I and type II diabetes men show disrupted sperm mitochondrial membrane potential, elevated intracellular ROS levels, and increased sperm DNA fragmentation, ultimately contributing to subfertility [129, 130, 131].

In women with diabetes, mitochondrial dysfunction in oocytes has been linked to reduced oocyte quality and abnormal oocyte mitochondria may be maternally transmitted to the embryo, persisting and propagating throughout embryogenesis and fetal development, potentially contributing to reproductive challenges in diabetic females [126, 127]. Further, diabetes‐induced OS and impaired mitochondrial bioenergetics can disrupt oocyte maturation, compromise fertilization potential, and impair embryo development [30]. Diabetic oocytes exhibit low ATP levels and reduced TCA cycle metabolites, leading to impaired mitochondrial function. Additionally, abnormal mitochondrial morphology and altered dynamics may disrupt chromosome congression during meiotic maturation [132]. Emerging evidence suggests that diabetes can alter the epigenetic landscape of oocytes, influencing gene expression patterns that are critical for embryonic development. Moreover, diabetes‐induced OS can damage mtDNA, leading to mutations that further impair mitochondrial function and trigger apoptosis [133]. In addition, it was reported that maternal diabetes‐induced mitochondrial impairments trigger cumulus cell apoptosis via cytochrome c release, disrupting oocyte‐supporting interactions and contributing to oocyte incompetence and poor pregnancy outcomes [132]. Embryos from diabetic mothers, despite exhibiting normal morphology, display reduced levels of proteins regulated by the PGC‐1α mitochondriogenic pathway during early gestation [134].

Further research is requisite to unravel the complex interplay between diabetes, mitochondrial dysfunction, and reproductive health. Developing targeted therapies that preserve mitochondrial integrity and function in gametes may hold immense promise for improving reproductive outcomes and alleviating the burden of infertility in individuals with diabetes.

#### 4.2.2 Endometriosis

Endometriosis is estrogen‐dependent inflammatory disorder, characterized by the growth of uterine‐like tissue outside the uterus [135], is linked to mitochondrial dysfunction in cumulus cells [136], potentially contributing to lessen retrievable oocytes, a lower oocyte maturity rate, and decreased numbers of available and high‐quality embryos [137, 138, 139]. While the exact molecular mechanisms by which endometriosis causes infertility are not fully understood, report suggests that mitochondrial dysfunction, including reduced mitochondrial mass and decreased membrane potential in human granulosa cells, is associated with lower estradiol (E2) levels, potentially resulting in reduced fertilization rates, impaired oocyte maturation, and diminished oocyte quality, ultimately compromising fertility [140]. Further, cardiolipin, a key phospholipid in mitochondrial membranes, is significantly reduced in oocyte mitochondria of endometriosis patients, potentially disrupting mitochondrial morphology and function [141]. An ultrastructural study of oocytes from patients with minimal or mild endometriosis confirmed impaired mitochondrial structure and reduced mtDNA copy numbers, likely resulting from disrupted cytoplasmic maturation [142] Moreover, increased ROS levels, often observed in the cellular environment of endometriosis patients triggers endoplasmic reticulum (ER) stress, promoting apoptosis and contributing to oocyte and ovarian dysfunction and contributing to infertility [143].

Therefore, mitochondrial dynamics and homeostasis may have a significant role in endometriosis‐based infertility, and addressing mitochondrial dysfunction in oocytes and cumulus cells represents a promising avenue for improving ART outcomes in women with endometriosis. Further research is needed to fully elucidate the intricate relationship between endometriosis, oocyte mitochondrial dysfunction, and infertility, paving the way for targeted therapies to improve fertility outcomes in this patient population.

#### 4.2.3 Polycystic Ovary Syndrome (PCOS)

PCOS, a common and complex endocrine disorder that is characterized by hyperandrogenism, irregular or absent menstrual periods (oligomenorrhea or amenorrhea), anovulatory cycles, and the presence of multiple ovarian cysts with hyperandrogenism [144]. The relationship between PCOS and infertility is multifaceted, with mitochondrial dysfunction in oocytes, granulosa cells, and cumulus cells emerging as a significant contributing factor [145]. Oxidative stress, inflammation, and hyperandrogenism in PCOS can contribute to mitochondrial dysfunction, affecting energy production, calcium homeostasis, and apoptosis regulation in reproductive cells [145, 146]. Though the precise mechanisms underlying mitochondrial dysfunction in PCOS oocytes are still not clear, factors such as insulin resistance [147] chronic inflammation, and hormonal imbalances [148] commonly associated with PCOS are thought to contribute to oxidative stress and mitochondrial damage within oocytes. Decreased mtDNA copy numbers and mutations in the mitochondrial genome, especially in mtRNA genes in PCOS patients, may relate to control of insulin resistance or other metabolic factors [149, 150].

Metabolomics analysis of follicular fluid indicates significant alterations in cellular metabolic pathways in classic PCOS patients, including upregulated glycolysis, a dysregulated TCA cycle, reduced LDH activity, and decreased NAD catabolism, potentially impacting the follicular and oocyte microenvironment [150]. Supporting the link between PCOS and mitochondrial dysfunction, studies have shown that growth hormone (GH) combined with gonadotropins significantly improves mitochondrial function in granulosa cells and oocyte quality [151], while mitochondrial‐targeted supplementation has been effective in restoring impaired mitochondrial function in PCOS women [152].

Therefore, targeting mitochondrial dysfunction in oocytes represents a promising avenue for improving fertility outcomes in women with PCOS. Further research is necessary to fully elucidate the complex interplay between PCOS, oocyte mitochondrial dysfunction, and infertility; paving the way for targeted therapies to enhance fertility outcomes in this patient population.

#### 4.2.4 Obesity

Obesity is recognized as a major health concern globally, and its impact on fertility is of increasing concern [153, 154]. Male obesity is associated with increased reactive oxygen species (ROS) and impaired mitochondrial function in sperm, both of which contribute to subfertility [155]. Obesity creates a state of metabolic stress that negatively impacts oocyte and cumulus cell mitochondrial function [85, 156] which elevates levels of ROS in the body and within oocytes, impairs oocyte maturation, induces early apoptosis, and alters epigenetic modifications, which may contribute to reduced oocyte quality [157]. This dysfunction manifests as ultrastructural defects, mitochondrial aggregation, reduced membrane potential, and increased intracellular calcium ion concentration [158, 159], which are strongly associated with impaired fertility.

Maternal obesity has been shown to significantly reduce the expression levels of key mitochondrial dynamics‐related proteins, such as DRP1 and MFN2, in oocytes [160]. Additional studies have reported decreased DRP1 activation accompanied by increased reactive oxygen species (ROS) production, collectively impairing mitochondrial dynamics and function [161]. Further, in obese mice model confirmed that enrichment of mitochondria‐associated ER membranes can elevate mitochondrial Ca^2+^ levels, a change that has been linked to increased apoptosis and impaired cytoplasmic maturation in oocytes [162]. In addition, obesity negatively affects fission and fusion processes of oocyte mitochondria, leading to fragmented mitochondria with reduced energy production and increased ROS generation [163]. Moreover, obesity disrupts the balance of mitochondrial biogenesis and mitophagy. This leads to an accumulation of dysfunctional mitochondria within oocytes, compromising their energy production capacity and overall function [163]. Interestingly, Phoenixin, a neuropeptide, has demonstrated potential in restoring mitochondrial dynamics in obese women, thereby enhancing oocyte quality and overall fertility outcomes [160].

Mitochondria‐targeted therapeutics have the potential to restore mitochondrial function in oocytes affected by obesity‐induced dysfunction [164]. Thus, addressing mitochondrial dysfunction in oocytes represents a potential strategy for improving fertility outcomes in obese women; more research is needed to develop targeted interventions that can effectively address obesity‐related infertility.

### 4.3 Mitochondrial Disease and Infertility

While mitochondrial dysfunction presents a significant challenge in reproductive medicine, impacting both male and female fertility, mitochondrial diseases are a group of genetic disorders caused by mutations in either nuclear DNA or mtDNA, affecting mitochondrial function. These diseases can manifest with a wide range of symptoms, depending on the severity of the mutation and the tissues affected. In the context of reproduction, mitochondrial diseases can directly impact reproductive health, leading to infertility in both men and women [165].

In men, mitochondrial dysfunction can impair spermatogenesis and sperm function, leading to reduced fertility [26]. Oxidative stress and mutations in the mitochondrial genome have been linked to decreased sperm motility, DNA fragmentation, and impaired fertilization capacity [166]. Mutations in mtDNA can disrupt oxidative phosphorylation, leading to ATP depletion and impaired sperm motility, morphology, and viability [167]. The deletions in mtDNA have been linked to oligoasthenospermia, a condition characterized by low sperm count and poor motility [166, 168]. Excessive ROS production due to functional defects of mitochondria can damage mitochondrial membranes, proteins, and mtDNA, further impairing their function [26]. In addition, mtDNA mutation related to impaired mitophagy can lead to the accumulation of dysfunctional mitochondria, negatively impacting sperm function [26].

As the consequences of mitochondrial dysfunction in sperm, insufficient ATP production impairs the sperm's ability to swim effectively, and can lead to structural abnormalities in sperm, hindering its journey to the egg [169, 170]. Thus, mitochondrial dysfunction is a significant contributor to male infertility, affecting various aspects of sperm function.

In women, mitochondrial diseases can have a profound impact on reproductive health, as they can affect oocyte quality, fertilization, and embryo development. The mitochondrial bottleneck phenomenon during oogenesis introduces an additional layer of complexity to the inheritance of mtDNA [171]. As oocytes mature, a stochastic segregation of mitochondria occurs, resulting in each daughter cell inheriting a random subset of the maternal mtDNA pool. This process has the potential to amplify the proportion of deleterious mtDNA mutations in the offspring, thereby increasing the risk of mitochondrial disease transmission [172]. The consequences of mitochondrial dysfunction in oocytes are far‐reaching, impacting various stages of reproduction, like meiotic spindle assembly, chromosome segregation, and fertilization [173]. This can result in aneuploidy in embryos, compromising their development and implantation potential, a leading cause of miscarriage and birth defects [174, 175].

Addressing mitochondrial dysfunction in infertility requires a multi‐faceted approach, targeting both the underlying genetic defects and the downstream consequences of impaired mitochondrial function. Mitochondrial replacement therapies are one of the main approaches available, though they raise complex ethical and legal considerations surrounding germline modification and genetic parenthood [176]. These techniques involve replacing the mitochondria in an affected woman's egg or embryo with healthy mitochondria from a donor [177]. Though significant technical hurdles remain in safely and effectively editing mtDNA in germ cells or embryos, the advent of CRISPR‐Cas9 technology holds potential for correcting mtDNA mutations directly [172]. While the field is rapidly evolving, with promising therapies on the horizon, addressing the ethical and societal implications of these technologies, particularly those involving germline modification, is paramount. A balanced approach, combining scientific advancements with ethical considerations, will pave the way for a future where mitochondrial diseases no longer hinder the dream of parenthood.

### 4.4 Aging and Reproductive Decline

Aging is a complex biological process that casts a long shadow on reproductive function in both sexes. At the heart of this decline lies the gradual deterioration of mitochondrial function, the cellular powerhouse essential for gamete development and embryonic competence [29]. As cells age, mtDNA becomes more susceptible to mutations due to the lack of healthy repair mechanisms compared to nuclear DNA. These accumulated mtDNA mutations can lead to impaired mitochondrial dynamism, impairment of the NADH/NAD+ redox, and reduced energy production over time [178]. Further, the capacity for mitochondrial biogenesis declines with age. This reduction in mitochondrial turnover results in a decrease in the number of functional mitochondria within cells, contributing to overall mitochondrial dysfunction [27]. Moreover, with aging, the balance between reactive oxygen species production and antioxidant defense mechanisms shifts, leading to increased oxidative stress and subsequent damage to mitochondrial proteins, lipids, and DNA [179]. This oxidative stress can further impair mitochondrial function and contribute to cellular senescence.

While males retain the capacity for spermatogenesis throughout life, aging subtly yet steadily chips away at sperm quality and fertility. This decline correlates strongly with accumulating damage to mtDNA within sperm cells [180]. Sperm, with their limited cytoplasmic space, are particularly vulnerable to oxidative stress, an imbalance between reactive oxygen species and antioxidant defenses [26]. Aging exacerbates this vulnerability, as mitochondria become less efficient at producing ATP, leading to increased ROS leakage and a vicious cycle of oxidative damage to mtDNA, proteins, and lipids [180]. The inner mitochondrial membrane potential, crucial for ATP synthesis, weakens with age, further compromising energy production and increasing ROS generation [26]. This energetic deficit manifests as reduced sperm motility, impaired capacitation, and a higher incidence of DNA fragmentation, ultimately hindering fertilization success [167]. Further, emerging evidence suggests that age‐related mitochondrial dysfunction extends beyond immediate cellular damage, influencing the epigenetic landscape of sperm [26].

Female fertility faces a steeper decline with age, largely attributed to the finite ovarian reserve and the oocyte's susceptibility to accumulating mitochondrial damage [29]. Oocytes heavily depend on mitochondrial biogenesis to meet the high energy demands of maturation, fertilization, and early embryonic development. However, this process becomes increasingly inefficient with age, leading to a decline in both the number and quality of mitochondria within oocytes [1]. The meiotic spindle, responsible for accurate chromosome segregation during oocyte maturation, is exquisitely sensitive to ATP fluctuations [28]. Aberrant DNA methylation patterns, potentially linked to impaired mitochondrial function, can be transmitted to offspring, impacting their long‐term health and potentially contributing to transgenerational effects of aging [28]. Age‐related mitochondrial dysfunction, with its accompanying energy deficits, increases the likelihood of spindle assembly errors, leading to aneuploidy in eggs, a major cause of miscarriage and birth defects [29].

Moreover, aging disrupts the delicate balance of mitochondrial dynamics, leading to an accumulation of fragmented, dysfunctional mitochondria within oocytes, further compromising their developmental competence [29]. The importance of balanced mitochondrial function is further underscored by the observation that maternal age‐related meiotic errors can be mitigated by reducing mitochondrial function. These errors are associated with impaired spindle assembly and altered kinetochore‐microtubule ratios, highlighting the intricate link between mitochondrial function and chromosome segregation during meiosis [60, 181]. Therefore, the relationship between mitochondrial function and embryonic development, particularly in the context of maternal age, is important. The mitochondrial respiratory function, as measured by oxygen consumption rate (OCR), increases alongside embryonic growth [182]. However, a decline in OCR can be observed at the morula stage in embryos from older mothers, and this suggests that the age‐related decline in mitochondrial function may contribute to the lower developmental rates observed in older women undergoing assisted reproduction. Thus, interventions aimed at improving mitochondrial function in aging oocytes could potentially improve IVF outcomes.

Age‐related infertility is a multifactorial process, with mitochondrial dysfunction playing a central role; understanding the intricate link between mitochondrial health and reproductive decline opens avenues for potential interventions. Lifestyle modifications, antioxidant therapies, and emerging mitochondrial rejuvenation strategies hold promise for preserving reproductive function and extending the window of reproductive opportunity.

## 5 Therapies Targeting Mitochondrial Metabolism in Reproductive Health

Given the crucial role of mitochondria in reproductive processes, researchers are actively exploring a variety of therapeutic strategies to target mitochondrial dysfunction and improve reproductive outcomes. These strategies can be broadly categorized into two main approaches: chemical interventions and cellular interventions.

### 5.1 Chemical Interventions

Antioxidant therapies aim to mitigate the damaging effects of oxidative stress on mitochondria [111]. These therapies involve supplementing with antioxidants that can scavenge ROS and protect cellular components from damage to some extent. Among the antioxidants that have been utilized, Coenzyme Q10 (CoQ10) [183], Carnitine [184], Resveratrol [185], Melatonin [186], Vitamins A, B, C, and E [187] have all shown potential benefits in improving oocyte and sperm quality by reducing oxidative stress and improving mitochondrial functions [1]. Coenzyme Q10 and carnitine have been associated with enhanced sperm motility and oocyte quality, while vitamin E and melatonin have shown potential in reducing oxidative stress and improving embryo development [183, 184, 185, 186, 187]. However, the evidence on the efficacy of these supplements in boosting fertility and pregnancy rates is still limited, and further research is needed to establish optimal dosages and understand their mechanisms of action in the context of human reproduction. These antioxidants have the potential to mitigate the damaging effects of OS on mitochondria, which can contribute to suboptimal reproductive outcomes. In addition, NAD+ boosting, through supplementation with NAD+ precursors, has emerged as a potential therapeutic strategy for ameliorating age‐related and metabolically induced infertility by enhancing mitochondrial function [188] and oocyte maturation competence [189]. While the preliminary evidence is encouraging, more research is needed to fully understand the efficacy and optimal dosages of these antioxidant therapies in improving fertility and pregnancy rates in clinical settings.

### 5.2 Cellular Interventions

Within the realm of cellular interventions aimed at enhancing reproductive outcomes, several innovative strategies have emerged that specifically target mitochondria within the female reproductive system. These interventions, primarily focused on optimizing oocyte quality and mitigating the transmission of mitochondrial disorders, encompass a range of techniques, including mitochondrial replacement therapy, mitochondrial transplantation, and the delivery of stem cell‐derived exosomes to sperm or testis, oocytes or ovaries. Each of these approaches holds unique potential for addressing mitochondrial dysfunction and improving reproductive outcomes in individuals facing infertility or carrying mtDNA mutations.

**FIGURE 4:**
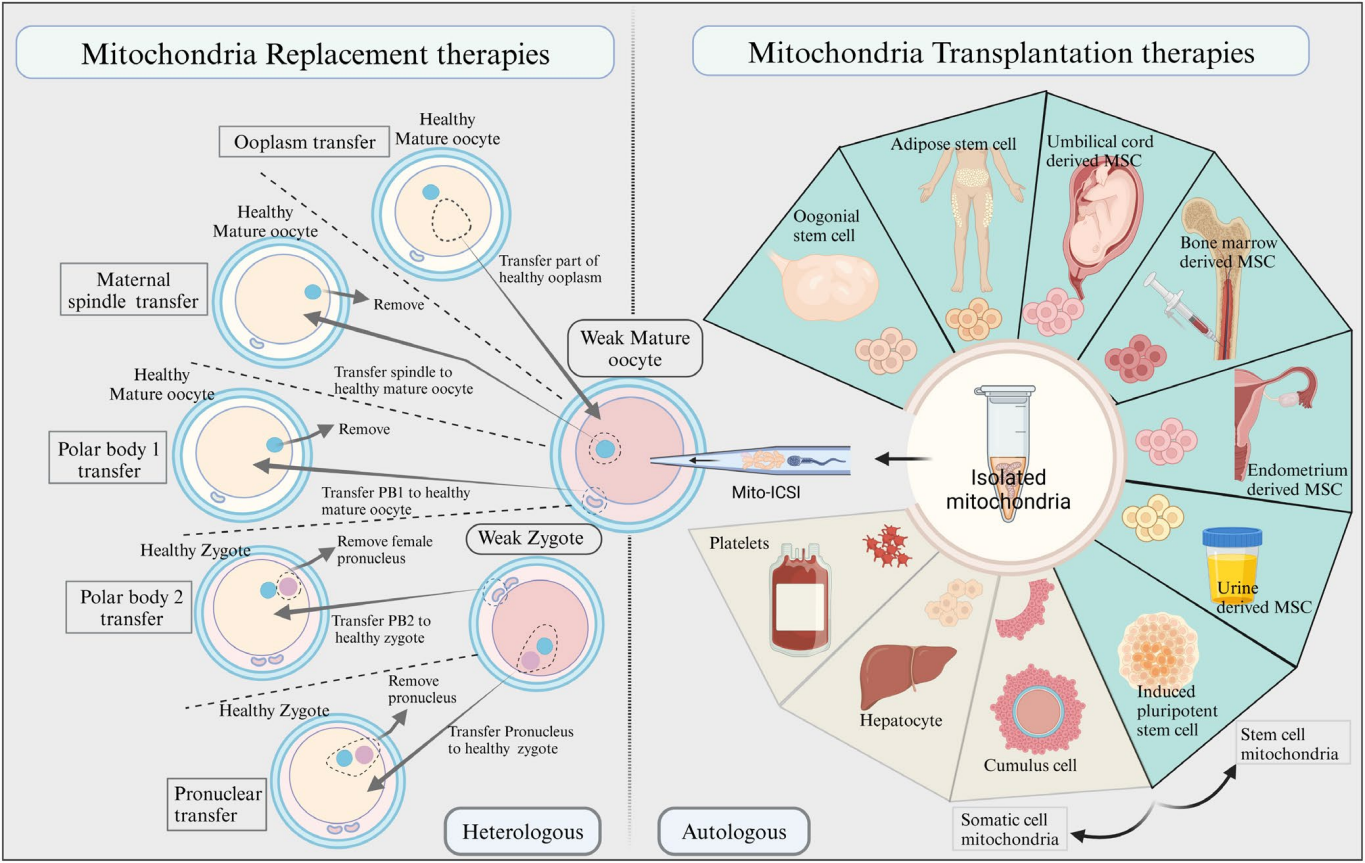
The primary cellular interventions aim to restore mitochondrial function in oocytes and early embryos: mitochondrial replacement therapy (MRT) and mitochondrial transplantation therapy (MTT). MRT encompasses several heterologous approaches, utilizing exogenous mitochondria. Conversely, MTT employs autologous mitochondria, with limited clinical and preclinical studies exploring both somatic cell and stem cell sources.

**Mitochondrial Replacement Therapies:** Mitochondrial replacement therapies (MRT) represent a groundbreaking advancement in assisted reproductive technologies, offering a potential solution for women who are holding risks of transmitting mitochondrial diseases to their offspring. These techniques involve replacing the mitochondria in an affected woman's oocyte or embryo with healthy mitochondria from a donor, thereby preventing the inheritance of debilitating or fatal mitochondrial disorders. While MRTs hold immense promise for families affected by these diseases, they also raise complex ethical and societal considerations surrounding germline modification and genetic parenthood (Figure 4 and Table 1). Careful consideration of the ethical, safety, and societal implications is indispensable to ensure the responsible development and application of this technology.

**TABLE 1:** Clinical applications of heterologous mitochondrial replacement therapies in oocytes.

Technique	Model	Outcomes	Limitations	References
OT	Mature oocyte	Successfully achieved live birth in patients with recurrent implantation failure	Concerns about the health status of offspring and mtDNA heterogeneity	[190]
	Mature oocyte	Higher fertilization rates and better embryo quality, in older women; healthy children born	Technical complexity; regulatory concerns regarding mitochondrial heteroplasmy	[191]
	Mature oocyte	No improvement in embryo quality or implantation using cryopreserved donor cytoplasm	Ineffective in older women with diminished ovarian reserve; high biochemical loss rate	[192]
GVT	GV oocyte	Survival rate of 77%, a fertilization rate of 52%, and a satisfactory early embryonic cleavage	Role of mitochondrial DNA of donor/recipient oocytes on the development and resulting embryos are unknown	[193]
	GV oocyte	No live birth or transferable embryo developments. Four out of five oocytes show normal maturation.	Microfilament disruption before enucleation is required	[194]
MST	Mature oocyte	Six newborns in 28 cycles, an increase in the maternal mtDNA haplotype	One in six newborn was heteroplasmic	[195]
	Mature oocyte	Efficient mtDNA replacement, Successful development of blastocysts	A significant higher abnormal fertilization rate in MST zygotes	[196]
PNT	Zygote	Early PNT performed 8 h after ICSI can achieve blastocyst formation rates at control levels, mtDNA carryover reduced to < 2% in 79% of oocytes	Only one baby born so far. No available data related to heteroplasmy	[197]
PB1T	Mature oocyte	The rate of blastocyst (42%) was lower compared to the control (75%). Pregnancy outcomes is not reported	Low and abnormal efficiency in cleavage. Lower blastocyst development rate compared to control, higher abnormal morphokinetics	[198]
	Mature oocyte	Form meiotic spindles and undergo successful fertilization, lower blastulation rate	Clinical data are limited, need Further optimization	[199]
PB2T	Human & mice oocyte	Successful production of embryos	Not enough clinical data available	[200]

*Note:* Six prominent mitochondrial replacement strategies are reviewed, summarizing their respective models, outcomes, and limitations.

#### 5.2.1 Ooplasmic Transfer

Ooplasmic transfer (OT) is an assisted reproductive technology first introduced in the late 1990s to improve the quality of oocytes with compromised function [190]. OT involves extracting a small amount (1%–5%) of cytoplasm, containing mitochondria and other essential molecules, from a healthy donor egg and injecting it into the recipient egg [190], with the aim of improving its quality and developmental potential. This procedure can be performed before or during the in vitro fertilization process. Though a report shows no beneficial outcome [192] most other studies suggested that OT could enhance oocyte fertilization, embryo development, and even lead to successful pregnancies in women who had previously experienced infertility due to poor egg quality [190, 191, 201].

However, despite its promising nature, OT has several drawbacks. A major concern is the potential for mitochondrial heteroplasmy, where offspring inherit mitochondrial DNA from both the donor and recipient, leading to unpredictable health outcomes [202, 203]. Additionally, OT raises ethical concerns regarding germline modification and the involvement of a third‐party's genetic material.

#### 5.2.2 Germinal Vesical Transfer

Germinal Vesicle Transfer (GVT) is a micromanipulation technique that involves transferring the nucleus (germinal vesicle or GV) of an immature oocyte, arrested in prophase I, into the cytoplasm of an enucleated recipient oocyte [193, 194]. This technique has potential applications in addressing both oocyte quality issues and mitochondrial diseases, which may help reduce the high rates of aneuploidy commonly seen in older women. An immature oocyte containing the GV is held in place, and the GV is aspirated along with a small amount of surrounding cytoplasm (karyoplast). A recipient oocyte, typically from a healthy donor, is enucleated, removing its own chromosomes and meiotic spindle; the karyoplast containing the GV is then injected into the perivitelline space of the enucleated recipient oocyte.

GVT can be used to improve the developmental competence of oocytes from women with advanced maternal age or other conditions associated with reduced oocyte quality [194]. By transferring the GV into a healthier cytoplasmic environment, the oocyte may gain access to essential factors and organelles, potentially improving its ability to mature and fertilize successfully [193, 194]. Also, GVT can be employed to prevent the transmission of mitochondrial diseases from mother to offspring. By transferring the GV of an oocyte from a woman carrying mtDNA mutations into an enucleated donor oocyte with healthy mitochondria, the resulting embryo will inherit the nuclear DNA of the intended mother and the healthy mtDNA of the donor [204].

The major challenge in GVT for preventing mitochondrial diseases is the potential carryover of some mutant mtDNA along with the GV and required extra technical skills [205]. Even small amounts of carried‐over mutant mtDNA can replicate and potentially cause disease in the offspring.

#### 5.2.3 Maternal Spindle Transfer

Maternal spindle transfer (MST) was another innovative technique in reproductive medicine designed to prevent the transmission of mitochondrial diseases from mother to child. It involves replacing the nucleus of an egg containing mutated mtDNA with the nucleus of a healthy donor egg, effectively replacing the diseased mitochondria. The meiotic spindle, containing the mother's nuclear DNA, is extracted from her oocyte, leaving behind the cytoplasm with mutated mitochondria. The mother's isolated spindle is transferred into the enucleated donor oocyte [206]. A novel MST approach has been developed using a mouse model, in which chromosomes are artificially aggregated into a single cluster before transfer. These aggregated chromosomes are then introduced into the cytoplasm of an enucleated oocyte, a method that may significantly reduce mtDNA carryover compared to traditional MST techniques [207].

Maternal spindle transfer offers women with mitochondrial diseases a path to having children free from these often‐debilitating conditions [208]. Though this technique has demonstrated high success rates, with studies reporting promising outcomes in fertilization, embryo development, and successful pregnancies [195, 209], some studies show higher abnormal fertilization [196]. Despite its potential benefits, maternal spindle transfer raises significant ethical concerns as it involves germline modification, and genetic alterations will be passed down to future generations, raising questions about informed consent and unforeseen long‐term consequences. Additionally, the involvement of genetic material from a third party, the egg donor, introduces complex legal and social questions regarding parentage and the rights of the child [210].

#### 5.2.4 Pro‐Nuclear Transfer

Pronuclear transfer (PNT) is a specialized assisted reproductive technology classified as another mitochondrial replacement therapy. It is designed to prevent the transmission of inherited mitochondrial disorders from a woman to her offspring [211]. PNT aims to replace the mother's mutated mtDNA with healthy mtDNA from a donor, thereby circumventing the transmission of the mitochondrial disease [197, 212, 213] and advanced maternal age and recurrent embryo arrest cases [214].

The PNT procedure involves a squarely orchestrated two‐step process performed shortly after in vitro fertilization. Initially, the pronuclei, containing the nuclear DNA (nDNA) of both the intended mother's oocyte (carrying the mutated mtDNA) and a donor oocyte (possessing healthy mtDNA), are carefully extracted. Subsequently, the intended parents' pronuclei are inserted into the enucleated donor oocyte, which houses the healthy mitochondria. This reconstructed oocyte effectively contains the nuclear genetic material of the intended parents and the cytoplasmic mtDNA of the donor. PNT has shown promising results in preclinical studies and has been used in a limited number of cases to achieve live births [212, 213, 215].

#### 5.2.5 Polar Body Transfer

Polar body transfer (PBT) is another reproductive technique designed to prevent the transmission of mitochondrial diseases from mother to child. It capitalizes on the natural process of meiosis, where an oocyte divides unequally to produce a mature egg and polar bodies. PBT involves transferring the nuclear genetic material from an oocyte carrying mutated mtDNA into a healthy donor oocyte that has had its own nuclear DNA removed.

Polar body transfer encompasses two main techniques: First Polar Body Transfer (PB1T) [198, 199, 216, 217, 218] and Second Polar Body Transfer (PB2T) [200, 219, 220]. In PB1T, mature oocytes are collected from both the intended mother and a healthy donor. The first polar body, carrying a near‐identical copy of the mother's nuclear DNA, is extracted from her oocyte after the first meiotic division. The donor oocyte is then enucleated, and the isolated first polar body is transferred into it. PB2T follows a similar procedure, but utilizes the second polar body, formed after fertilization, which contains a haploid copy of the mother's nuclear DNA. Polar body transfer presents several potential advantages as a mitochondrial replacement technique. Studies indicate that PBT may lead to lower levels of carryover of the mother's original mtDNA compared to other techniques, potentially minimizing the risk of disease transmission [204]. Since the second polar body is naturally enclosed by the nuclear membrane, its removal and subsequent injection into enucleated oocytes or zygotes is relatively straightforward [215]. Furthermore, some argue that PBT raises fewer ethical concerns compared to methods like pronuclear transfer because it utilizes the polar body, a byproduct of natural conception that is typically discarded [221].

Despite its potential, polar body transfer presents several challenges. The technique is highly complex, requiring specialized equipment and expertise [204]. Another concern is the potential for PBT to disrupt genomic imprinting, a process where specific genes are expressed differently depending on parental origin [204, 222]. While PBT represents a promising approach for preventing the transmission of mitochondrial diseases, further research is requisite for optimizing the technique before widespread clinical implementation.

**Mitochondria Transplantation Therapy (MTT):** Mitochondrial transplantation therapy encompasses a spectrum of innovative approaches designed to ameliorate mitochondrial dysfunction by introducing healthy mitochondria into oocytes exhibiting compromised mitochondrial function. MTT strategies primarily diverge in the cell source of mitochondria and the specific oocyte developmental stage targeted for intervention. At present, many scientists from different countries have explored various stem and somatic cell types as mitochondrial donors, with recipient oocytes at various stages, including mature metaphase II and immature germinal vesicle stages (Figure 4).

#### 5.2.6 Clinical Approaches of Autologous Stem Cell Mitochondria Transplantation to Oocyte

Among autologous stem cell, oogonial stem cells (OSC), purportedly collected from the ovarian cortex, represent the only stem cell‐derived mitochondria utilized in clinical applications. Such treatments have been reported in several countries, including Canada, the United Arab Emirates [223], Turkey [224], Spain [225], and Japan [226].

While most of the studies have reported positive impacts on embryo development and implantation rates, the study conducted in Spain found no significant enhancement following OSC‐derived mitochondrial transplantation into oocytes [225]. A Japanese research group reported that OSC‐derived mitochondrial transplantation improved embryo quality and live birth rates, and their subsequent follow‐up study on offspring (age 2–4 years) born from this procedure revealed no significant developmental defects [226]. The study resulted in 13 live births, and analysis confirmed that the babies' mitochondrial DNA was primarily derived from their mothers, indicating the safety of the procedure [226].

Despite the potential of OSC‐derived mitochondria as a viable source for transplantation, uncertainties persist regarding the definitive existence of OSCs and their isolation protocols [227]. Furthermore, the substantial physical and financial burdens imposed on patients undergoing such treatments have hindered the widespread adoption of OSCs as a mitochondrial source for transplantation. In addition, there have been attempts to use mitochondria from bone marrow‐derived mesenchymal stem cells [228] and urine‐derived mesenchymal stem cells [229] for transplantation; these approaches are limited and not yet considered feasible approaches (Table 2).

**TABLE 2:** Clinical approaches of autologous stem cell mitochondria transplantation to oocyte.

Mitochondria source	Model and stage	Outcomes	Limitations	References
Oogonial stem cell	Human, Mature oocyte	Significantly higher good embryo rate	Invasive, resting time to next cycle, high cost, retrospective study	[224]
Oogonial stem cell	Human, Mature oocyte	Significantly improved fertilization rates with a trend for better embryo grades	Invasive, resting time to next cycle, high cost, retrospective study	[223]
Oogonial stem cell	Human, Mature oocyte	No significant improvement in embryo development and live birth rate	Invasive, resting time to next cycle, high cost, retrospective study	[225]
Oogonial stem cell	Human, Mature oocyte	Significantly higher good‐quality embryo development (6.9% vs. 23.7%) and live birth rate (0% vs. 17.5%), no birth defects	Invasive, resting time to next cycle, high cost, retrospective study	[226]
Bone marrow derived MSC	Human, Mature oocyte	Significantly higher good‐quality embryo than that in previous cycles (33.17% vs. 0%, *p* < 0.05)	Invasive, retrospective study	[228]
Urine derived MSC	Human, Mature oocyte	Increase mitochondrial content, activity, and cytoplasmic Ca2+, improvements in embryonic morphological indicators, euploidy rates	No clear data related to embryo development and pregnancy	[229]

*Note:* Six clinical trials have explored autologous stem cell mitochondrial transplantation in oocytes. Four trials utilized oogonial stem cells as the mitochondrial source, while the remaining two employed bone marrow‐derived and urine‐derived mesenchymal stem cells, respectively.

#### 5.2.7 Clinical and Pre‐Clinical Autologous Somatic Cell Mitochondrial Transfer

While a limited number of studies have explored the feasibility of transplanting somatic cell mitochondria into oocytes to enhance oocyte quality, several challenges persist. One mouse study demonstrated that microinjecting mitochondria from liver cells into aged oocytes failed to improve the low fertilization and embryonic development rates [230]. The authors concluded that mitochondria derived from liver cells and potentially other somatic cell types might function differently within oocytes.

Another study investigated the transfer of autologous cumulus cell and granulosa cell mitochondria into compromised oocytes within the ICSI procedure. This study reported that the procedure led to a decreased propensity for rapid oocyte cleavage, reduced apoptosis and fragmentation rates, improved fertilization rates, a lower abortion rate, and increased live birth rates [231]. Further, another research explored the use of platelet mitochondria, revealing improvements in mitochondrial morphology, ATP production, and blastocyst development rates when young autologous mitochondria were introduced into aged oocytes (Table 3) [232].

**TABLE 3:** Preclinical and clinical applications of somatic cell mitochondrial transplantation to oocytes.

Mitochondria source	Model	Outcomes	Limitations	References
Cumulus cell	Human, aged, mature oocyte	Significant higher pregnancy rate, lower abortion	Possibility of somatic cell mtDNA mutations transfer	[231]
Hepatic cell	Mice, aged and young mature oocyte	Fertilization rate and blastulation rate were not improved	Somatic cell mtDNA mutations transfer	[230]
Platelets	Hamsters, aged and young mature oocyte	Fertilization rate and blastulation rate were not improved	Possibility of somatic cell mtDNA mutations transfer	[232]

*Note:* Three distinct somatic cell types were utilized as mitochondrial donors. No beneficial outcomes were reported.

However, significant concerns remain regarding the use of somatic cell mitochondria. The potential for accumulated mutations within somatic cell mtDNA raises the possibility of transmitting these mutations to offspring, potentially resulting in heteroplasmic negative influences. Consequently, somatic cell mitochondrial transplantation is not currently considered a viable or potential approach for clinical applications.

#### 5.2.8 Preclinical Approaches for Autologous Stem Cell Mitochondrial Transfer

A growing body of research, primarily conducted in animal models, has explored various cell types as potential sources of healthy mitochondria for transplantation. Autologous stem cell mitochondrial transfer is an emerging therapeutic approach that offers a significant advantage by minimizing the risk of immune rejection associated with using donor material; it is being investigated for its potential to address mitochondrial dysfunction and improve reproductive outcomes in animals. This technique involves isolating mitochondria from a female's own stem cells, typically mesenchymal stem cells due to their accessibility and abundance, and transferring them into her oocytes to enhance their quality and developmental competence.

Adipose‐derived stem cells (ASC), known for their accessibility and ease of isolation, have shown promise as a source of mitochondria for improving oocyte quality in preclinical studies [233, 234]. Similarly, umbilical cord‐derived mesenchymal stem cells, a readily accessible postnatal source, have also been investigated as a potential source of mitochondria for MTT. It was reported that umbilical cord stem cell‐derived mitochondria can enhance oocyte quality and developmental competence in animal models [235, 236]. In addition, other stem cell sources, including bone marrow‐derived stem cells [228], urine‐derived stem cells [229], and endometrial stem cells [237], have also been explored as potential mitochondrial donors in animal models. These studies have collectively demonstrated the potential of mesenchymal stem cell‐derived mitochondria to improve oocyte quality and support early embryonic development in preclinical settings. Furthermore, induced pluripotent stem cells (iPSCs), which offer the advantage of patient‐specific derivation, have emerged as a promising avenue for personalized MTT [238, 239]. These advancements hold significant potential for developing patient‐tailored MTT strategies (Table 4).

**TABLE 4:** Preclinical approaches of autologous stem cell mitochondria transplantation to oocyte.

Mitochondria source	Model and stage	Outcomes	Disadvantage	References
Umbilical cord‐derived mesenchymal stem cells	Mice GV oocyte	Increase oocyte maturation rate, increase good embryo rate, increase live birth rate	Zona‐pellucida should be weaken, only good for who has previous deliveries	[236]
Endometrial mesenchymal stem cells	Mice mature oocyte	Significantly higher blastulation rate, live birth rate in aged mice	Invasive, have to wait to recover for embryo transfer	[237]
Adipose stem cell	Aged mice, mature oocyte	Significantly improved embryo development, increased live birth rate	Small samples size	[234]
Adipose stem cell	Mice, mature cryopreserved oocyte	Significantly improved embryo development, higher ATP production, lower ROS	Small samples size	[233]
Adipose stem cell	Aged mice, mature oocyte	ATP is significantly increased, blastulation rate not significantly increased	Small sample size	[240]
Adipose stem cell	Mice, mature cryopreserved oocyte, transgenerational analysis	Three generations bone after adipose stem cell mitochondria transferred show no any defect compared to wild type animals	No specific behavioral analysis	[241]
Induced pluripotent stem cell	Aged mice, Zygote	High blastulation rate, implantation rate, live birth rate in aged mice	Cell source not applicable for regenerative medicine. High tech expertise is required	[238]

*Note:* Various stem cell types have served as mitochondrial donors in preclinical models. These studies largely confirm the positive impact of stem cell mitochondrial transplantation. Notably, several groups have employed adipose‐derived stem cells and demonstrated the transgenerational safety of ASC‐derived mitochondria.

**FIGURE 5:**
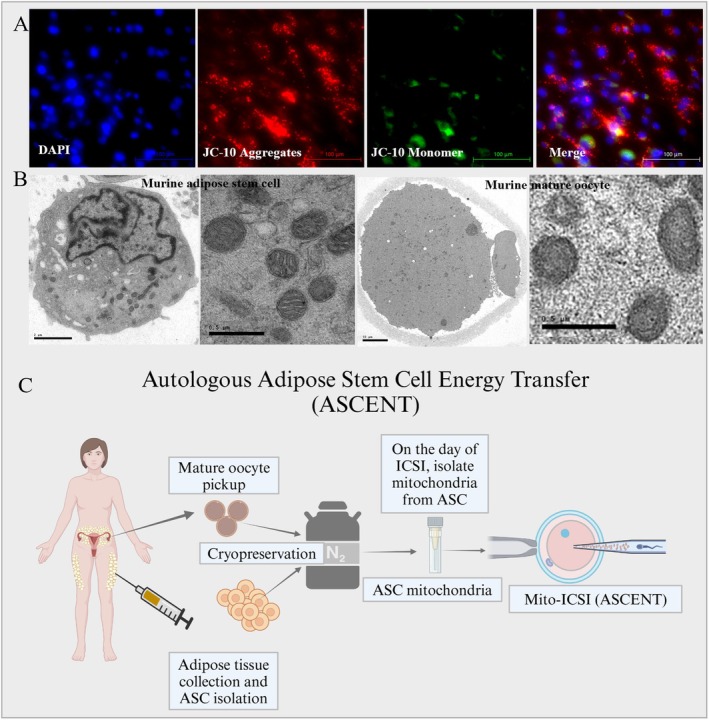
Adipose stem cell autologous mitochondrial transplantation therapy may be a promising strategy for enhancing oocyte quality. (A) Murine ASCs exhibit higher mitochondrial membrane potential [233] (B) Mitochondria isolated from murine ASCs display an oval morphology with limited cristae, resembling those observed in mature murine oocytes [233]. (C) Schematic illustration for prospective adipose‐derived stem cell energy transfer (ASCENT) procedure.

Additionally, adipose derived MSC or ASC are emerging as a promising source for regenerative medicine due to their unique properties, including their ability to differentiate into various cell types and their abundance in readily accessible adipose tissue [242]. While ASCs have shown promise in various applications, recent research suggests that their regenerative potential that significantly linked to the functionality of their mitochondria. Moreover, studies indicate that ASCs exhibit robust mitochondrial function, characterized by higher mitochondrial membrane potential and lower ROS levels compared to other cell types, such as endometrial stem cells [241, 243, 244]. This superior mitochondrial function translates to increased energy production, which is crucial for the energy‐intensive processes of cell proliferation, differentiation, and tissue regeneration [245, 246]. And ASCs mitochondria are morphologically resembled with oocyte mitochondria (Figure 5A,B) [233, 240] and cell itself known to secrete a diverse array of growth factors and cytokines that promote tissue repair and regeneration [247]. Adipose tissue is readily available and easily obtainable through minimally invasive procedures compared to other sources like bone marrow [248]. ASCs exhibit immunomodulatory capabilities, suppressing excessive immune responses and creating a favorable microenvironment for tissue regeneration [249]. The relative ease of isolation, expansion, and potential for autologous transplantation make ASCs a clinically attractive option for regenerative therapies [242].

In regards to reproductive therapies, some studies have shown that transplantation of ASC‐derived mitochondria can improve oocyte quality in aged mice and cryopreserved oocytes, enhance embryo development, and increase pregnancy rates [233, 234]. This study demonstrated that transplantation of adipose‐derived stem cell (ASC) mitochondria enhanced the ATP production capacity of embryos without increasing ROS levels, likely due to improved mitochondrial dynamics and function following the transplantation [233]. These findings highlight the potential of ASC mitochondria as an effective therapeutic strategy for addressing mitochondrial dysfunction in reproductive aging and infertility.

With the promising potential of clinical application, in recent study the research demonstrates that ASC MTT enhances the developmental competence of oocytes in mice, leading to improved fertilization and embryo development. Importantly, the study found no significant adverse effects on the health, behavior, or reproductive performance of offspring across three generations [241]; this study termed as prospectively as Adipose Stem Cell‐derived Mitochondria ENergy Transfer (ASCENT, Figure 5C). Among available autologous stem cell MTT studies at preclinical level, ASCENT might be a safe and promising strategy for enhancing the quality of oocytes, especially in cases of advanced maternal age or other conditions that compromise oocyte quality [233, 234].

While animal studies have provided valuable insights into the feasibility and potential benefits of MTT, translating these findings into safe and effective clinical applications remains paramount. Rigorous preclinical studies are crucial to thoroughly assess the long‐term safety and efficacy of MTT [250]. Further, rigorous long‐term follow‐up is necessary to assess the safety and efficacy of MTT and monitor for any potential adverse effects in offspring [251]. Therefore, further research is warranted to translate these promising findings into clinical applications and fully elucidate the potential of ASC mitochondria for regenerative medicine, including in the context of reproductive health [252]. Addressing ethical considerations and establishing standardized protocols for mitochondrial sourcing, characterization, and techniques of transplantation are essential steps toward advancing MTT into clinical practice [253].

## 6 Ethical and Legal Considerations in Mitochondrial Therapies

Mitochondrial replacement therapies and transplantation therapies (MRT/MTT), while promising for treating mitochondrial diseases and infertility, raise complex ethical and legal considerations that necessitate careful scrutiny and ongoing societal dialogue. A significant ethical concern surrounding MRT, particularly techniques like pronuclear transfer and spindle transfer, is that they result in germline modifications that are passed down to future generations [176]. Additionally, the involvement of mitochondrial DNA from a third party complicates the traditional understanding of genetic parenthood, potentially leading to legal disputes and psychological implications for families [210].

While preclinical studies have shown promise, the long‐term health effects of MRT/MTT on offspring remain largely unknown [177, 254]. Concerns exist regarding potential incompatibilities between nuclear and mitochondrial DNA from different sources, which could lead to unforeseen health issues later in life [255]. Rigorous long‐term follow‐up studies are crucial to assess the safety and efficacy of these techniques before widespread clinical implementation. The complexity and cost of MRT/MTT raise concerns about equitable access. Without careful consideration, these therapies could exacerbate existing health disparities, with only affluent individuals able to afford these potentially life‐altering treatments. Ensuring equitable access to these technologies requires careful policy development and resource allocation.

Given the complexity and novelty of MRT/MTT, ensuring comprehensive informed consent from prospective parents is paramount [256]. This involves clear communication of the potential benefits, risks, and uncertainties associated with the procedure, as well as the potential implications for future generations. Access to genetic counseling is essential to support informed decision‐making. The rapidly evolving nature of MTT necessitates robust regulatory frameworks to ensure responsible development and application [257]. Striking a balance between fostering scientific progress and safeguarding ethical principles requires ongoing evaluation and revision of existing regulations. The possibility of unforeseen complications, such as immune rejection or the introduction of harmful mitochondrial mutations, necessitates rigorous preclinical and clinical evaluation. Addressing ethical considerations requires a multidisciplinary approach, involving open dialogue between scientists, clinicians, ethicists, policymakers, and the public. Establishing robust regulatory frameworks, ensuring transparency and informed consent, and fostering ongoing societal debate are crucial for navigating the ethical complexities of mitochondrial transplantation in reproductive medicine.

## 7 Future Potentials of Mitochondria Related Therapies

Mitochondrial transplantation offers significant potential for treating mitochondrial dysfunction, but further advancements are needed to ensure safe and effective clinical application. Refining transfer methods like microinjection is essential to enhance efficiency and reduce cellular damage [258]. Identifying optimal sources of healthy mitochondria is also critical. Though still in early stages, these strategies may transform infertility treatment, prevent mitochondrial disease transmission, and counter reproductive aging.

Various protocols exist for mitochondrial isolation, with differential centrifugation being the most widely used due to its simplicity. However, the mechanical stress involved can compromise mitochondrial membrane integrity and function. Ensuring the functional quality of isolated mitochondria remains a key challenge, highlighting the need for gentler yet effective isolation techniques.

Although mitochondrial transplantation into oocytes has shown promise, little is known about how transplanted mitochondria integrate into the host environment. A better understanding of this adaptation is crucial for ensuring the safety and effectiveness of clinical applications. While current findings are promising, further research is needed to clarify how mitochondrial transfer affects reproductive outcomes and to enhance the safety and efficiency of these methods. Although autologous transfer is preferred, it is not always feasible, and allogeneic approaches raise ethical and logistical concerns. Research should focus on donor matching, standardized sourcing protocols, and quality control. Post‐transfer, the integration and functionality of donor mitochondria are vital for success. Future studies should explore ways to enhance mitochondrial incorporation, support biogenesis, and maintain long‐term function within recipient cells [259].

Although mitochondrial transfer has mainly focused on female infertility, exploring regenerative strategies to enhance mitochondrial function in male reproductive cells is equally important. Evidence from ischemic cardiomyopathy models shows that adipose‐derived stem cells (ASCs) can transfer functional mitochondria to damaged myocardial cells [246]. This supports the regenerative potential of ASCs and highlights their promise for treating male infertility linked to mitochondrial dysfunction.

The use of donor mitochondria, especially in germline therapies, presents significant ethical and legal challenges related to informed consent, genetic parenthood, and long‐term effects on offspring. Addressing these concerns requires ongoing societal dialogue and the development of clear regulatory frameworks to ensure responsible use. Balancing scientific advancement with ethical integrity and patient safety is essential for the future of mitochondrial therapies.

Mitochondrial transplantation represents a transformative approach to treating reproductive disorders linked to mitochondrial dysfunction [63]. While challenges remain, continued research and innovation offer hope for clinical translation, particularly for infertility and mitochondrial diseases. ASC‐derived mitochondria show strong potential as an accessible and effective source for enhancing oocyte quality.

However, ethical and legal concerns, especially around germline modification and genetic parenthood, must be addressed [176]. Moving forward, transparency, inclusivity, and a strong emphasis on long‐term safety and societal impact will be essential to responsibly advance these therapies.

## 8 Conclusion

Mitochondria play a vital role in both male and female reproduction, with their number, dynamics, and function precisely regulated in a cell‐specific manner. Disruptions to this balance can impair key reproductive processes. In women, mitochondrial function is essential for oogenesis, oocyte maturation, fertilization, and early embryonic development. In men, mitochondria are crucial for spermatogenesis, sperm motility, and fertilization capacity. Mitochondrial dysfunction, whether from genetic mutations, environmental stressors, or aging, can significantly reduce fertility in both sexes.

Emerging evidence indicates that improving mitochondrial health may be a promising strategy for treating infertility. Lifestyle changes that enhance mitochondrial biogenesis and reduce oxidative stress have shown modest benefits; however, more targeted interventions are needed. Mitochondrial transplantation therapy (MTT) offers a novel approach by replacing damaged mitochondria in oocytes or embryos. Although still in early development, preclinical studies in animal models suggest its feasibility and potential. Further research is essential to ensure safe and effective clinical application in humans.

Future research should aim to refine MTT protocols by identifying optimal mitochondrial sources, standardizing isolation and transfer methods, and evaluating long‐term safety and efficacy. Advancements in mitochondrial‐targeted therapies such as gene editing to correct mtDNA mutations or drugs that boost mitochondrial biogenesis also offer promising avenues. Recognizing the pivotal role of mitochondria in reproduction and investing in the development of innovative mitochondrial and mitochondrial‐targeted therapies is of fundamental importance for improving fertility outcomes and expanding treatment options for individuals and couples facing infertility.

## Conflicts of Interest

The authors declare no conflicts of interest.
